# Formulation and Evaluation of Eudragit RL-100 Nanoparticles Loaded In-Situ Forming Gel for Intranasal Delivery of Rivastigmine

**DOI:** 10.15171/apb.2020.003

**Published:** 2019-12-11

**Authors:** Sara Salatin, Jaleh Barar, Mohammad Barzegar-Jalali, Khosro Adibkia, Mitra Alami-Milani, Mitra Jelvehgari

**Affiliations:** ^1^Research Center for Pharmaceutical Nanotechnology Biomedicine Institute, Tabriz University of Medical Sciences, Tabriz, Iran.; ^2^Student Research Committee, Tabriz University of Medical Sciences, Tabriz, Iran.; ^3^Department of Pharmaceutics, Faculty of Pharmacy, Tabriz University of Medical Sciences, Tabriz, Iran.; ^4^Drug Applied Research Center, Tabriz University of Medical Sciences, Tabriz, Iran.

**Keywords:** Cytotoxicity, Eudragit, Hydrogel, Nanoparticle, Nasal, Rivastigmine

## Abstract

***Purpose:*** Rivastigmine hydrogen tartrate (RHT) is commonly used for the treatment of mild to moderate Alzheimer’s disease (AD). The aim of this work was to formulate in-situ pluronic F-127 (PF-127) hydrogels containing Eudragit RL-100 (EU-RL) nanoparticles (NPs) in order to improve the therapeutic efficacy of RHT through the nasal route.

***Methods:*** The NPs were prepared using different polymer to drug ratios and evaluated for their physicochemical characteristics, cellular uptake and in vitro cytotoxicity against lung adenocarcinoma cells (A459). PF-127 nanoformulations were prepared via cold method and analyzed in terms of physicochemical properties and drug release profiles. The nanoformulations and plain drug gel were then assessed by ex vivo permeation studies across the sheep nasal mucosa.

***Results:*** The EU-RL NPs exhibited a particle size within the range of 118 to 154 nm and positive zeta potential values of 22.5 to 30 mV with an approximately spherical shape. Fourier transform infrared spectroscopy (FTIR), differential scanning calorimetry (DSC), and X-ray powder diffraction (XRPD) suggested no drug to polymer interaction through the preparation of nanoformulations. The RHT-loaded NPs exhibited an acceptable cytocompatibility with a time- and dose-dependent cellular internalization.

***Conclusion:*** Our results clearly indicated the potential of nanoformulations as controlled release systems to improve the therapeutic efficacy of RHT through the intranasal administration

## Introduction

Alzheimer’s disease (AD) is a progressive neurological disease thatseriously affects older adults.^1^ Rivastigmine hydrogen tartrate (RHT) is applied to the treatment of AD and acts as a reversible inhibitor of anticholinesterase and butyrylcholinesterase.^2^ Currently, this drug is on the market for oral administration as purposes including capsule and tablet dosage forms. However, oral delivery of RHT has severallimitations such as first pass liver metabolism and destruction of the drug by digestive enzymes or the acidic pH of the stomach, resulting in low bioavailability.^3,4^ On the other hand, drug transport into the brain is significantly blocked by the existence of the blood-brain barrier.^5^

Mucosal surfaces have been widely investigated as a potential administration routefor the therapeutic agents with poor oral bioavailability.^6,7^ Among those,the intranasal route has been demonstrated as an effective technique for direct drug delivery to the brain which may bypass the blood-brain barrier.^8^ Besides, concentration-time profiles of drugs following nasal administration are comparable to those achieved after the intravenous delivery.^9^ The therapeutic agents can be absorbed across the mucous membrane in the olfactory region of the nasal cavity and be reached directly into the brain via the cribriform plate.^10,11^

Polymeric nanoparticles (NPs) have great benefit for direct nose-to-brain drug delivery. Eudragit RL-100 (EU-RL) is selected as a positively charged polymer to prepare mucoadhesive NPs as it increases the interaction between mucin and NPs, thus drug bioavailability is increased.^12^

Aqueous solutions are not suitable as intranasal formulations because of the fast-occurring clearance of the nasal cavity which may result in loss of therapeutic effect. However, a thermoreversible hydrogel formulation of drug is more feasible and convenient for the intranasal medication, as it is a liquid at room temperature and turns into a gel at body temperature.^13^ PF-127 can be applied to prepare the in-situ hydrogel forming systems.^14^ PF-127 aqueous solution within a certain range of concentration forms a liquid at room or lower temperatures, but spontaneously forms a non-flowing gel at body temperature.^15^

The present study aimed to develop thermoreversible gel formulations embedded with EU-RL NPs for the intranasal delivery of RHT, using PF-127 as a gelling agent. The nanoformulations were evaluated in terms of gelation temperature, viscosity, pH, mucoadhesion strength, stability and release studies. The cellular uptake and *in vitro* cytotoxicity of NPs against lung carcinoma cells (A459) were evaluated. The permeability of nanoformulations was also addressed through the sheep nasal mucosa.

## Materials and Methods

### Materials

EU-RL was provided by Akbarie Co. (from RÖhm Pharma GMBh, Weiterstadt, Germany). RHT was kindly provided by Tofigh-Daru (Tehran, Iran). PF-127 (molecular weight of 9840-14 600) was purchased from Sigma-Aldrich (St. Louis, USA). For cell culture tests, RPMI-1640 Medium, 3-(4, 5-dimethylthiazol-2-yl) 2, 5-diphenyltetrazolium bromide (MTT) and Fluorescein isothiocyanate (FITC) from Sigma-Aldrich (Poole, UK), Human lung adenocarcinoma cell line (A549) from the national cell bank (Tehran, Iran) and fetal bovine serum (FBS) from GIBCO/Invitrogen (Paisley, UK) were obtained. Dialysis membrane (mol wt cut off10 000-12 000 Da) was supplied by Biogen (Mashhad, Iran). Phosphate buffered saline (PBS) and all other chemicals and solvents were of analytical grade. Deionized water was used throughout the study.

### Preparation of RHT NPs

The RHT NPs were formulated using different polymer to drug ratios through the nanoprecipitation technique.^16^ RHT (35 mg) and EU-RL in different weight ratios (Table 1) were dissolved in water (2 mL) and acetone (5 mL), respectively. The RHT solution was added dropwise into the EU-RL organic solutionunder vigorous stirring at 400 rpm. The obtained organic solution was then injected into the external solution (10 mL of 3% PF-127). Acetone was completely evaporated under stirring at room temperature. The prepared NPs were subjected to centrifugation (60 minutes at 18 500*×*g), washed and freeze-dried. The above method was also followed to prepare blank NPs.

**Table 1:** Evaluation of characteristics of RHT NPs and nanoformulations, Comparison of various release characteristics, flux and permeability coefficient of RHT from different nanoformulations and plain drug gel

**Variables**	**Formulation code**			
	**Characterization of nanoparticles**			
	N0^a^	N1	N2	N3
EU-RL/RHT ratio	-	4:1	7:1	10:1
EU-RL (%w/w)	-	80	87.5	90.91
*Drug entrapped (% ± SD)	-	38.40 ± 8.94	56.20 ± 1.86	62.00 ± 2.78
Zeta Potential (mV)	-	+22.5	+30.8	+26.8
Particle size (nm)	-	118	123.9	154
**Appearance**	**Characterization ofnanoformulations**			
	**Transparent**	**Turbid**	**Turbid**	**Turbid**
*T_sol-gel_ (°C ± SD)	28 ± 0.121	29 ± 0.101	30 ± 0.047	32 ± 0.191
*pH ( ± SD)	6.4 ± 0.21	6.1 ± 0.058	5.8 ± 0.013	5.8 ± 0.082
*Loading capacity (% ± SD)	0.00 ± 1.00	1.98 ± 0.01	2.17 ± 0.03	2.43 ± 0.03
*Loading efficiency (% ± SD)	100 ± 0.10	95.19 ± 0.33	102.73 ± 2.13	104.51 ± 2.44
Viscosity (cP ± SD)	132 ± 0.17	130 ± 0.23	122 ± 0.101	118 ± 0.23
Mucoadhesive strength (N/cm^2^ ± SD)	47 ± 0.85	69.02 ± 3.68	63.34 ± 12.97	58.95 ± 5.59
Rel_0.5_ (%)^b^	25.88 ± 1.20	44.84 ± 0.00	33.29 ± 0.74	28.44 ± 1.36
Rel_24_ (%)^c^	102.94 ± 0.94	81.73 ± 0.58	73.41 ± 2.63	65.36 ± 1.24
DE	56.35 ± 0.19	74.30 ± 0.66	65.95 ± 2.33	58.44 ± 0.51
MDT (min)	131.82 ± 23.93	130.99 ± 8.63	146.39 ± 14.38	152.39 ± 15.36
f_1_^d^	0	33.62 ± 1.49	16.33 ± 2.39	7.55 ± 0.88
Flux^e^ (mg/cm^2^.min) *10^-3^	24 ± 5.10	49 ± 5.29	44 ± 1.00	16 ± 5.10
^*^K^f^_p_ (cm/min) *10^-4^	16 ± 3.60	34 ± 1.40	30 ± 0.68	11 ± 3.50

EU-RL, Eudragit RL100; DE, dissolution efficiency; MDT, mean dissolution time; ^a^Plain drug gel (with untreated RHT (rivastigmine hydrogen tartrate)); ^b^Amount of drug release after 0.5 h; ^c^Amount of drug release after 24 h; ^d^Difference factor (used to compare multipoint dissolution profiles) (0<f1<15); ^e^Amount of drug passed through unit of surface area; ^f^permeability coefficient; *P< 0.05. All tests were performed three times (n=3).

### Characterization of NPs

#### Size and zeta potential

A dynamic light scattering (DLS) (Malvern, UK) was used to evaluate the mean diameter and zeta potential of the fresh RHT NPs. For this, the prepared nanosuspensions were diluted with deionized water and sonicated for 5 minutes prior to analysis.

### Preparation of plain drug gel

PF-127 solution was prepared according to the usual “cold method” developed by Schmolka.^17^ For this, PF-127 (1.8 g) were slowly added into 10 mL chilled deionized water (4°C) under magnetic stirring. The RHT powder (10 mg) was added to the clear solution containing 18% PF-127 (10 mL) and gently mixed with the magnetic stirrer. The prepared solution was then stored in a refrigerator at 4°C.

### Preparation of gel system containing RHT NPs

The required weight of PF-127 granules was dispersed in the RHT nanosuspensions (10 %w/v) and stirred until a clear solution was formed (Table 1).

### Characterization of gel basedsystems

#### Morphological analysis

Scanning electronmicroscopy(SEM, MIRA3 TESCAN, Czech Republic) was utilized to determine the morphological features of NPs incorporated into the hydrogel. The specimens were placed on double-sidedcarbon stickytape on a SEMstub and covered with platinum/palladium coatings under vacuum prior to inspection by electron microscope.

#### Loading capacity and loading efficiency

The prepared RHT nanoformulations (1 mL) were transferred into glass vials containing 5 ml acetone and shaken at room temperature (for about 24 h) to obtain a clear solution. The resulting solutions were then filtered and measured at 263.4 nm using a UV-Visible spectrophotometer (UV-160 Shimadzu, Japan).Loading capacity (LC) and loading efficiency (LE) of nanoformulations were examined by the following equations:

LC (%) = A/B ×100

LE (%) = A/C ×100

Where A is the total amount of drug incorporated into the nanoformulations, B is the amount of nanoformulations used, and C is initial drug content of NPs initially taken to prepare nanoformulations.

#### Sol-to-gel transition temperature

The transition temperature from sol to gel (T_sol-gel_) was measured over a range of temperature (5-45°C). For this, 5 mL of samples was filled into glass vials, placed into a shaker incubator and stirred at 100rpm. Temperature was slowly increased and sol-to-gel temperatureof the nanoformulations measured upon inversion of the vials.^18^ The gelling temperature range suitable for the intranasal gels is 30-34°C.

#### Viscosity

Inorder to address the effect of NPs’ loading on the hydrogel viscosity, the viscosity measurements of the plain drug gel and nanoformulations were performed by a rotational viscosimeter (Pro model, Brookfield with LV spindle 2, Middleboro, MA, USA) at a rotation rate of 50 rpm, when samples were in sol state at 25 ± 2C.^19^

#### pH

All pH measurements were individually made with a calibrated metrohm 827 pH meter after diluting small aliquots of the nanoformulations into deionized water (1:10 (w/v)).

#### Mucoadhesive strength

Themucoadhesive strength of each nanoformulation and the plain drug gel was examined using the freshly excised nasal mucosa of sheep. A modified balance technique was used for the measurement of the force required for the separation of samples from the mucosal tissue.^20^ The left pan of a double-pan balance was removed and a glass vial hanged inversely with yarn. Another glass vial was placed below the upper vial. The lower vial was filled with PBS and hold at 32-34°C temperature. The nasal mucosa was attached to the surface of the lower vial with adhesive tape. The prepared gel was then placed on the mucosal tissue and both vials connected together for 2 minutes. Next, a number of weights were put on the right pan of the balance until the upper vial separated from the nasal mucosa.

#### Stability

The stability of nanoformulation (N3) was tested for appearance, pH measurement, sol-gel transition temperature and loading capacity in different storage conditions including 2-8°C, 25°C/60% relative humidity (RH), and 40˚C/75% RH for a period of 3 months.^21^ The analysis of drug concentration was performed on a Knauer smartline-HPLC (Berlin, Germany) containing a pump (Smartline 1000) and a UV/Visdetector 2500. HPLC separation was made by a C_18_-column (4.6 mm×250 mm, 5 μm) using a mobile phase of 90% v/v methanol and 10% v/v water at a flow rate of 2.0 mL/min. The detection of drug was performed at room temperature at 211 nm.

#### Fourier transform infrared spectroscopy (FTIR)

The FTIRmeasurements of RHT powder, EU-RL, PF-127, physical mixture, blank and developed nanoformulations were performed with the assistance of a computerized apparatus (Bruker, Tensor 27, USA) operating in the 400-4000 cm^-1^ wave number range at resolution 1 cm^-1^.

#### Differential scanning calorimetry (DSC)

The thermal properties were tested by DSC (Shimadzu, Japan). Samples (2 mg) were placed in aluminum pans and heated from 25 to 300˚C at 10˚C/minheating rate.

#### X-ray powder diffractometry (XRPD)

The scanning of all samples was performed using XRPD (Bruker Axs, D8 Advance diffractometer) at a scan speed of 2˚C/min with the 2θ range of 10-90˚C.

#### Drug release

The RHTrelease from the plain drug gel and nanoformulations was studied using the dialysis bag diffusion technique.^22^ To this end, the RHT nanoformulations (1 g, gel state) were placed in the dialysis bags containing nasal simulated fluid (NSF, 1 mL). Dialysis membranes were then immersed in vials filled with PBS (25 mL), placed in a shaker incubator and shaken at 100 rpm for 24 hours at32 ± 1˚C. In regular time intervals, the aliquots of release medium were sampled and replaced with an equal quantity of fresh media. Finally, all samples were filtered and analyzed using a UV spectrophotometer at the wavelength of 263.4 nm.

To exhibit the differences between formulations, a mathematical comparison was carried out using differential factor (*f*_1_) by the following equation:

$$f_{1}=([\sum_{t=1}^{n}R_{t}-T_{t}]/[\sum_{t=1}^{n}R_{t}])\times 100$$

Where R_t_ is the percentage dissolved of the formulation at time t, T_t_ is the percentage dissolved of the formulation in comparison with time t and n is the number of dissolution sample time points.

#### Release kinetics

In order to investigate the kinetic of release of RHT from the prepared nanoformulations, data of drug release was fitted into the various kinetic models like Higuchi, Hixson Crowell, zero order, first order and Korsemeyer-Peppas models.

### Cellular studies

#### Cytotoxicity assay

The cytotoxicity of pure RHT, RHT-loaded NPs and empty NPs was tested against A549 cells using the MTT assayprotocol.^23^ For this, the cells were seeded into plastic 96-well plates and incubated with complete RPMI 1640 culture medium (with 10% FBS, 100 U/mL penicillin and 100 μg/μL streptomycin) in a humidified incubator (37°C, 5% C02). After overnight incubation, cells were treated with treatment compounds in six different concentrations containing 50, 100, 250, 500, 750 and 1000 μg/mL of RHT or respected concentration of the loaded and empty NPs. Moreover, a 5% dimethylsulfoxide (DMSO) was considered as a positive control and cells without any treatment served as a negative control. Cell viability was determined after incubation for 24 and 48 h. For this, cell culture media was replaced by 150 μL fresh complete medium and 50 μL MTT reagent (2 mg/ml in PBS) and plates incubated for about 4 h. The medium plus MTT was then replaced with 25 μL Sorenson’s glycine buffer and 200 μL of DMSO due to solubilize formazan crystals. A microplate spectrophotometer/reader (ELX 800, Biotek, CA. USA) was used to determine the optical density of each well at 570 nm.

#### Flow cytometry

The cellular uptake of EU-RL NPs was quantified using flow cytometric analysis. FITC-labeled NPs were fabricated by a nanoprecipitation methodlike the ones mentioned above where FITC used in place of RHT and free FITC removed from the NPs by high-speed centrifugation. A549 cells were seeded into six-well plates and cultivated until confluence. The cell culture media was removed, replaced by fresh medium containing 100 and 200 µg/mL of FITC-labeled NPs and incubated with cells (2 h at 37˚C). The cells were collected by trypsinization and diluted in cell-culture medium. After centrifugation, cells were re-suspended in 500 µl PBS and analyzed using the flow cytometer.

#### Fluorescence microscopy

Cells (A549) were seeded (at a final concentration of 4×10^5^ cells per well) onto glass cover slips in a six-well plates. After a 24 h incubation, the growth medium in the wells was replaced with fresh medium containing 100 µg/mL of FITC-labeled NPs and incubated (1 and 2 h at 37˚C). At every time interval, the supernatants were discarded and cells rinsed with PBS. The cellular uptake of NPs was then examined using a fluorescence microscope (Olympus, BX-50).

### Mucosal permeability

The permeability study was performed through the sheep nasal mucosa using Franz diffusion cells. The nasal tissues were mounted in the diffusion cells between the donor and receptor chambers. The receptor compartment was filled with 25 mL PBS and stirred at 100 rpm.^24,25^ Subsequently, 1 g of nanoformulations was placed on the mucosa and soaked in NSF. Both compartments were clamped together at 32°C. At predetermined time points until 8 h, the samples were collected and filtered using the 0.22 µm pore size filters. The amount of permeated drug was analyzed at 263.4 nm.

The drug permeability coefficient (K_p_) was determined using the following equation:

$$K_{P}=\frac{J_{ss}}{C_{0}}$$

Where K_p_ is the permeability coefficient of a drug in a particular vehicle (cm h^-1^), J_ss_ is the steady state permeation flux per unit area and C_0_ is the solute concentration in the donor compartment.

### Statistical analysis

All of the data were examined by *t* test and reported significant when *P*< 0.05.

## Results and Discussion

Gel system containing RHT NPs were prepared in order to increase contact time with the nasal mucosa and elevate the accessibility of rivastigmine to the brain region.

### Preparation of RHT NPs

Here, RHT NPs were successfully formulated using different polymer to drugratios based on the nanoprecipitation technique with some modification. The nanoprecipitation is the most suitable method to prepare NPs, avoiding the application of toxic solvents and surfactants.^26^ A major advantage of this method is the use of water miscible solvents that leads to an enough diffusion rate due to produce spontaneous emulsification.^27^ In this methodology, an aqueous solution is added dropwise using a syringe into an organic solution containing the polymer (EU-RL) with vigorous stirring until turbidity indicative of polymer precipitation is visually observed. NPs are created as a result of the acetone diffusion in water and rapid precipitation of the polymer forms the NPs.

### Characterization of NPs

#### Size, zeta potential and morphology

The particle size and surface charge dictate the biological fate of the particulate carriers. The average size of NPs was observed in the range of 118 to 158 nm as measured by DLS analysis (Table 1).An increase in the values of particles size and zeta potential was found by increasing the concentration of EU-RL used in the organic phase. Zeta potential is known as an important index of the stability of colloidal dispersions. As zeta potential is increased, the stability and monodispersity of the particles are increased irrespective of the charge type. This effect can be attributed to the high electric repulsion. The zeta potential values were positive (22.5 to 30.8 mV) for all the NPs (Table 1). However, different stability mechanisms of the NPs have been exhibited as a function of the adsorbed amount of surfactant molecules, indicating the existence of different conformations of the pluronic molecules in the adsorbed layer.^28^

**Figure 1:**
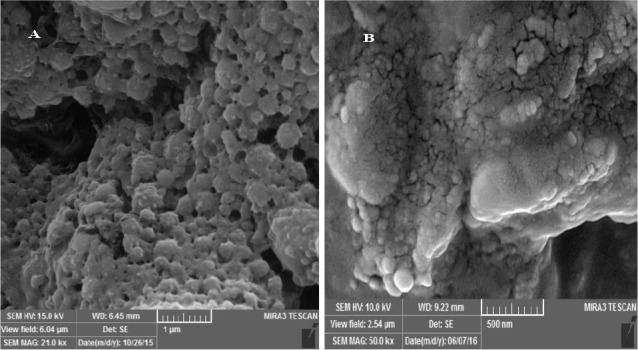
SEM images of the lyophilized EU-RL NPs (A), and EU-RL NPs embedded in gel (B).

The size and morphology of the free RHT NPs and RHT NPs incorporated into the hydrogel were characterized using SEM. Free NPs were found to be almost spherical in shape with a rough surface and could keep their perfect structures in hydrogel system, as shown in Figure 1.

### Preparation of Gel System Containing RHT NPs

The plain drug gel and RHT NPs-loaded gels were prepared using cold method, successfully. During the optimization of PF-127 ratio, a concentration of 18% (w/v) of PF-127 was recognized optimum for the incorporation of RHT-loaded NPs.

### Characterization of gel system

#### Loading capacity

Loading capacity of the nanoformulations (N1 to N3) with regards to the quantity of polymer (EU-RL) in the NPs varied from 1.98% to 2.43%, which was within the required limits. As a result, the nanoformulation N3 with 1:10 drug to polymer ratio had the highest loading capacity (Table 1). After comparing formulations, the best formulation was chosen and used for the extra studies.

#### pH

Sincean acidic or alkaline pH may damage mucous membrane of the nose, the determination of the pH value is an important parameter in designing mucoadhesive dosage forms. The pH of nanoformulations was ranged from 5.8 to 6.1 which was close to the nasal mucosal pH (*i.e.* 5.0 to 6.5) (Table 1).^29^ Therefore, the prepared nanoformulations may not result any significant damage on the nasal mucosa.

#### Appearance

The RHT nanoformulations were observed to be turbidbecause of the presence of fine colloidal NPs when compared with the plain gel (Table 1).

#### Sol-to-gel transition temperature

The determination of sol-to-gel transition temperature is a crucial step in the development of in-situ gelling formulations. The concentration of gelling polymer should be such that the formulation was in liquid state prior to the administration and transformed into gel once it acquired the in-situ temperature. Normally, an appropriate range of gelation temperature for the thermoreversible nasal gels would be 32-34°C. At a lower gelation temperature than 25°C,gelation takes place at room temperature which leads to the problems in manufacturing,handlingand administration. Consequently, if a thermogelling formulation is not getting gelled at a temperature below 34°C, it will stay in a liquid state at body temperature, resulting in a higher rate of clearance of dosage form.

PF-127has excellent thermosensitive gelling propertiesbecause of the negative solubility coefficient of block copolymer micelles.^30^ In general, the number of micelles is increased with an increase in the temperature, leading to the immobility of thesolution and gel formation.The T_sol–gel_ values obtained for the plain drug gel (N0) and nanoformulations (N1, N2 and N3) (*P*< 0.05) were shown in Table 1. Through this study, we observed that concentrations of 18% w/v of PF-127 were required to obtain a final nanoformulation with the transition behavior at 32-34°C. At higher gelation temperature, gel formation occurs at the lower PF-127 concentration range. Here, the incorporation of NPs into the hydrogel increased the sol-to-gel transition temperature which may probability be attributed to the low chance of packing of the PF-127 micelles in the presence of polymeric NPs.

#### Viscosity

The study of viscosity plays an importance role on the characteristics of hydrogel based formulations like spreadability and ease of syringe ability.^31^ To this end, we studied the effect of NP concentration (%w/w) on the viscosity for different prepared nanoformulations at25±2^°^C in sol state (Table 1). The viscosity of nanoformulations was found to be within the range between 130±0.12 to 118±0.23 cP. In comparison with the plain gel, the viscosity of the nanoformulations was low at all studied formulations, proportional to the concentration of the polymer and reduced slightly with increasing the concentration of EU-RL NPs. It seems that the presence of polymeric NPs can weaken hydrogen bond strength between PF-127 molecules and change many properties of the hydrogels embedded with NPs, e.g. by increasing gelling temperature/time or by decreasing viscosity.^32^

#### Mucoadhesive strength

The prepared plain gel and nanoformulations were checked for their mucoadhesive strengths bymeasuringthe forceneededto separate two surfaces after adhesion from each other. Here, the mucoadhesive strength increased (58.95 ± 5.09 to 69.96 ± 3.68 N/cm^2^), as the concentration of EU-RL polymer increased probably due to the electrostatic interactions between the EU-RL polymer and the mucosal layer (Table 1).

#### Stability

No significant change was observed in the quality characteristics of the nanoformulation upon 3 month storage at 2-8˚C. However, it was unstable at 25°C (60% RH) and 40°C (75% RH) (Table 2). High temperature conditions may cause the partial melting and thermodynamic instability of the EU-RL, resulting in aggregation. Besides, the physicochemical characteristics (gelation temperature, drug content and appearance) of the nanoformulation remained stable (*P*< 0.05) at 2-8˚C upon 3 months storage. Therefore, the NPs-loaded gel is proposed to be stored at 2-8°C.

**Table 2:** Physicochemical stability of the nanoformulation (N3) at different storage conditions

**Variables**	**Preliminary specifications**	**First month**			**Second month**			**Third month**		
		**2-8°C**	**25°C/60% RH**	**40°C/75%** **RH**	**2-8°C**	**25°C/60% RH**	**40°C/75% RH**	**2-8°C**	**25°C/60%** **RH**	**40°C/75% RH**
pH (n)	5.8	5.8	5.6	5.4	5.7	5.5	5	5.5	5.1	4.3
Turbidity	+	+	+	++	+	++	+++	++	+++	+++
Loading capacity (%±SD)	2.22	2.20±0.07	2.22±0.04	2.07±0.11	2.03±0.14	2.19±0.05	1.95±0.15	2.07±0.07	2.02±0.09	1.86±0.03
T_sol-gel_ (°C)^a^	32	32	28	25	33	25	25	33	25	25

RH, relative humidity. ^+^Less turbid gel; ^++^Turbid gel; ^+++^Very turbid gel; ^a^ Sol-gel transition temperature.

#### FTIR analysis

**Figure 2:**
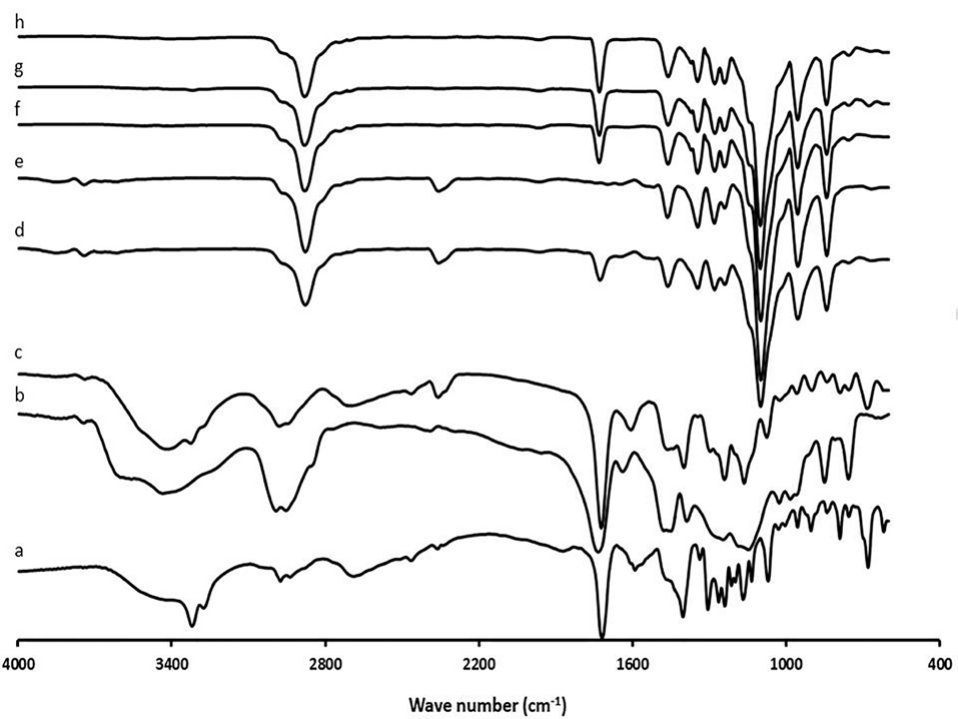
FTIR spectra of RHT (a), EU-RL (b), physical mixture N3 (c), blank N3 (d), PF-127 (e), N1 (f), N2 (g), and N3 (h).

On the basis of the data given in Figure 2, the absorption peaks were detected for the RHT at around 2974 cm^-1^ (CH3 antisymmetric stretching), 2850-2880 cm^-1^ (CH3 symmetric stretching), 1719.25 cm^-1^ (C=O), 3067-3093 cm^-1^ (C–H stretching in the benzene ring) and 1145.70-1590.19 cm^-1^ (C–H stretching in-plane bending). For the EU-RL spectrum, the strong bands were easily recognized in the regions between 1150-1190 cm^–1^ and 1240-1270 cm^–1^ due to the stretching vibration of the carbonyl from the ester groups of the polymer. Absorbance bands at 1734.01 cm^–1^ and 3437.91 cm^-1^ clarified the presence of C (=O) ester and associated OH groups, respectively.^33^ The FTIR spectrum of the PF-127 exhibited characteristic absorption peaks at 2885.42 cm^-1^ (C-H), 1112.89 cm^-1^ (C-O) and 1355.86 cm^-1^ (in-plane O-H bend).^34^ The physical mixture demonstrated that there is no chemical interaction between the EU-RL NPs and the drug molecules. For nanoformulations, the stretching band of the ester group C-O at 1070 cm^-1^, bending vibrations of -C-H at 1403 cm^-1^, stretching band of the carbonyl group at 1719 cm^-1^ and C-H stretching band at 2974 cm^-1^were recognized, showing the disappearance of most peaks in the nanoformulations in comparison with the pure drug.

#### DSC analysis

**Figure 3:**
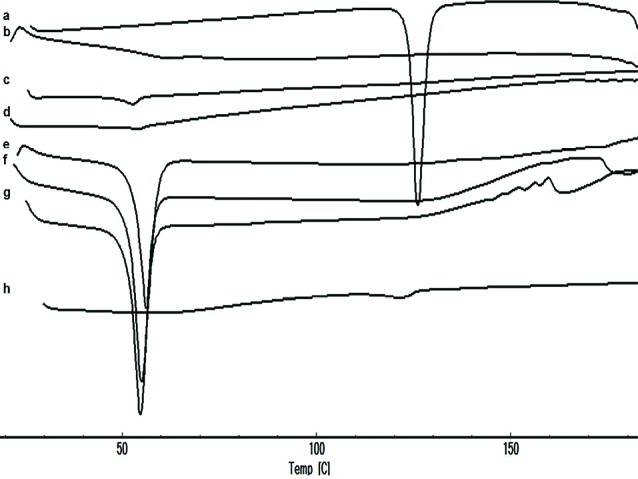
DSC thermograms of RHT (a), EU-RL (b), PF-127 (c), blank N3 (d), N1 (e), N2 (f), N3 (g), and physical mixture N3 (h).

According to the results shown in Figure 3, RHT exhibited a sharp peak at 126.22°C due to the anhydrous crystal form of the drug. The DSC peak of the EU-RL polymer showed its amorphous nature and no endothermic peaks were seen. PF-127 exhibited a melting endotherm peak at 52.76˚C. Besides, the thermogram of the physical mixture showed a low intensity of the melting endotherm of the RHT due to the dilution effect of the EU-RL. The DSC thermograms of nanoformulations did not exhibit peak near the RHT melting point, suggesting that the drug was diluted by the polymer into the NPs, because in NPs (1:4, 1:7 and 1:10 drug to polymer ratios) the amount of polymer was higher than the drug.

#### XRPD Analysis

**Figure 4:**
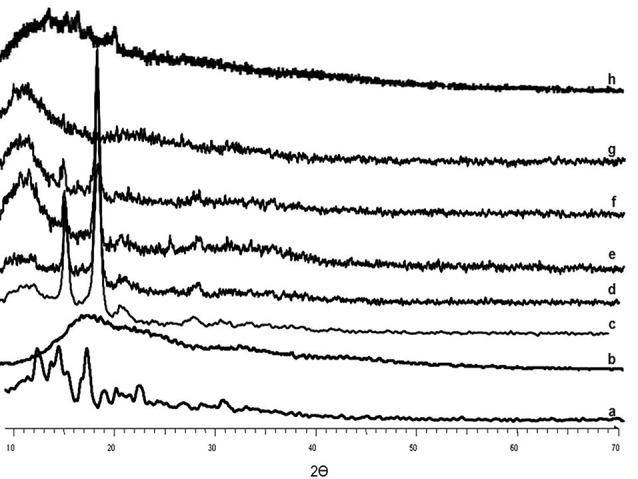
XRPD patterns of RHT (a), EU-RL (b), PF-127 (c), blank N3 (d), N1 (e), N2 (f), N3 (g), and physical mixture N3 (h).

The pure RHT presented sharp peaks at diffraction angles (2θ) of 9.6°, 11.4°, 13.4°, 14.2°, 15.7°, 19.2°, 20.2°, 22.4°, 24.8°, 26.8°, 29.6°, 31.3° and 33.7° which clearly confirm the crystalline nature of the drug, whereas diffractogram of the EU-RL showed an amorphous structure without obvious peaks (Figure 4). PF-127 displayed a crystalline structure and manifested a number of peaks at 13°, 18.5°, 23°, 26°, 35.5°, 39° and 43°. The physical mixture diffractogram exhibited low signal intensity of the drug due to the dilution effect of EU-RL. Furthermore, the XRPD pattern of the prepared nanoformulations did not show any dominant 2θ peak which might be attributed to the drug incorporation into the NPs.

#### Drug release

The profile of drug release from the all nanoformulations was characterized at 32°C for 24 h using the dialysis bag diffusion method. Cumulative percentage of drug release ranged between 60%-80% after 24 h for all nanoformulations. An initial burst release was measured in all three nanoformulations within the 0.5 h, followed by a sustained release. Further, the process of drug release was observed to slow down with the incorporation of the RHT-loaded NPs into PF-127 gel when compared with the plain gel.The reason is that during the *in vitro* release process,the drug loaded in NPs must be diffused into the hydrogel and then be diffused through the bulk framework of hydrogel. These findings suggest that RHT released for formulations N1, N2 and N3 within 24 h was 81.73%, 73.41% and 65.35%, respectively, while for the plain gel it was 102.94% within the first hours of drug release (Table 1).

The regression coefficients (R^2^) for N1, N2 and N3 nanoformulations were found to be 0.999, 0.947 and 0.919, respectively. Therefore, the Peppas model showed the highest correlation. These results suggest that drug release may mainly be controlled by diffusion process.

### Cellular studies

#### Cytotoxicity assay

**Figure 5:**
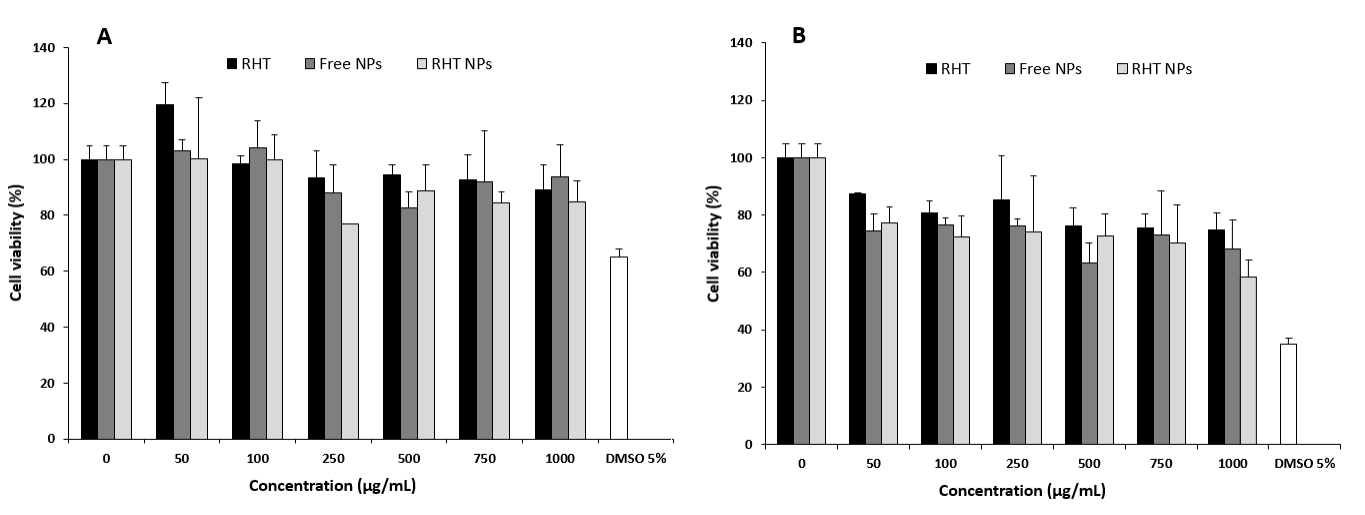
Cell viability of A549 cells after 24 (A), and 48 h (B) in response to various concentrations of the treatments. Each point is an average of four replications and the vertical bars represent standard deviations.

In order to address the cytocompatibility of RHT, empty and RHT NPs, the cell viability was tested using the MTT assay (Figure 5). A dose- and time-dependent cytotoxic effect against A549 cells was obtained by all studied compounds. These results indicate that the direct NP-cell surface interaction may lead to increased toxicity. In high doses, the pure drug was found to have growth inhibition activity at both incubation times (i.e. 24 and 48 h). However, the slight changes in the percentage of cell viability were observed at high concentrations of the treatments after 24 h. In comparison, the highest cytotoxicity effects of RHT NPs were resulted after 48 h incubation periods (*P*< 0.05) in a dose- and time-dependent manner. It was similarly investigated that the viability of Caco-2 cell line decreased at high doses (as mg/mL) of RHT.^35^ The low cytotoxic effect of the NPs on A549 cell line indicates the low risk of adverse effects and therefore the suitability of the prepared particles as a drug delivery system.

#### Flow cytometry

**Figure 6:**
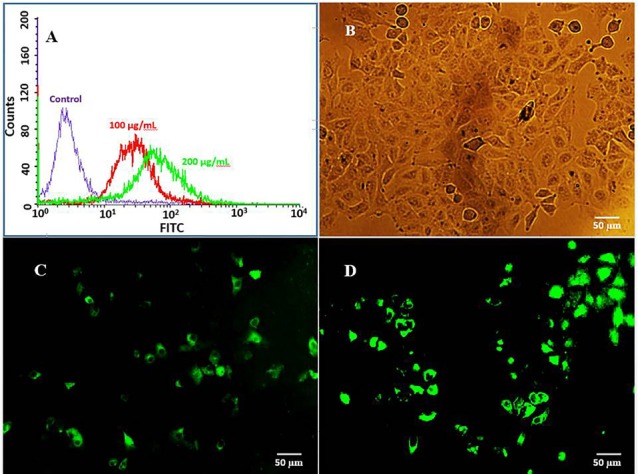
Cellular uptake of FITC-labeled EU-RL NPs analyzed by flow cytometry (A). Notes: A549 cells were treated with 100 and 200 (µg/mL) of FITC/EU-RL NPs for 2 h, and fluorescence intensity was measured by flow cytometry. Fluorescence microscopy analysis of cellular uptake of FITC/EU-RL NPs in A549 cells. Notes: Untreated control A549 cells (B), A549 cells were treated for 1h (C), and 2h (D) with 100 μg/mL of FITC/EU-RL NPs. Green fluorescence: FITC/EU-RL NPs.

The cellular uptake was shown by encapsulating FITC in the EU-RL NPs and fluorescent intensity for different samples analyzed by flow cytometry analysis. A peak shift to the right was observed for the histograms of FITC/EU-RL NPs, suggesting an efficient uptake of NPs by A549 cells (Figure 6A). A dose-dependent increase in the fluorescence intensity was observed from 100 up to 200 μg/mL.

#### Fluorescence microscopy

The cell incubation with 100 µg/mL of NPs for just 2 h showed a high percentage of fluorescent cells in media. No detectable cytotoxic effectwas observed on cell viability. The cellular uptake of FITC-labeled NPs was highly time-dependent and NPs internalized more efficiently after 2 h of incubation time (Figure 6C and D). This event can show the effect of particle size and surface charge on the cellular uptake of NPs. Previous studies have also suggested that the positively charged NPs show an increased cellular uptake when compared to the negatively charged NPs.^36,37^

### Mucosal permeability

The prepared nanoformulations were evaluated for drug permeationthrough the sheep nasal mucosa using Franz diffusion cells. Previous studies have revealed that pluronics reduce the drug release rate owing to their micelle structure. As illustrated in Table 1, the amount of drug permeated per unit area after 8 h was found to fall between 16×10^-4^ to 49×10^-4^ mg/cm^2^.min. The permeability constant, K_p_ (cm/min), was also estimated and found to be 34×10^-4^, 30×10^-4^ and 11×10^-4^ for N1, N2 and N3, respectively. RHT nanoformulations exhibited a significantly (*P*< 0.05) higher permeability than that of plain drug gel (i.e. flux, 24×10^-4^ mg/cm^2^.min and K_p_, 16×10^-4^ cm/min). The penetration of RHT through the mucosa is therefore highly influenced by the mucoadhesive property of EU-RL NPs. Increase in the RHT penetration through the nasal mucosa can also be related to the relatively large absorption surface area and it seems that NPs tend to accumulate in the mucosa and submucosa layers. This creates a reservoir of drug which is slowly released into the nasal area.^4^ Moreover, other studies providing evidence that particle size affects the absorption of NPs by the nasal tissue.^38,39^

## Conclusion

The purpose of the present work was to examine the advantages of EU-RL NPs embedded within PF-127 hydrogels as intranasal carriers. The prepared nanoformulations were evaluated in terms of appearance, pH, viscosity, sol-gel temperature, content and mucoadhesion strength. The RHT NPs were roughly spherical in shape with an average size of 118 to 154 nm. The RHT nanoformulations exhibited a transition temperature closer to the body temperatureand a sustained release profile over a 24 h period, mainly governed by Fickian diffusion. The RHT NPs demonstrated a low cytotoxic effect on the A549 cells which were uptaken by those in a dose- and time-dependent manner. The *ex vivo* permeation test showed an increased permeability of RHT across the nasal mucosa. The results from our study indicated the potential use of current nanoformulations as nasal drug delivery systems. Further studies are recommended to investigate the uptake mechanism of RHT NPs in the nasal epithelial cells, biodistribution as well as *in vivo* fate ofNPs in detail.

## Ethical Issues

This project has been approved by the Ethics Committee, with code number TBZMED.REC.1394.141.

## Conflict of Interest

The authors declare no conflicts of interest.

## Acknowledgments

The authors want to thank the authorities of the Research Council of Tabriz University of Medical Sciences for their financial support (grant no. 110) as a PhD thesis. The Student Research Center is also greatly acknowledged for a partial grant-in-aid (no. 59557).
